# Edc3 Function in Yeast and Mammals Is Modulated by Interaction with NAD-Related Compounds

**DOI:** 10.1534/g3.114.010470

**Published:** 2014-02-05

**Authors:** Robert W. Walters, Igor A. Shumilin, Je-Hyun Yoon, Wladek Minor, Roy Parker

**Affiliations:** * Department Chemistry and Biochemistry, University of Colorado, Boulder, Colorado; † Howard Hughes Medical Institute, University of Colorado, Boulder, Colorado; ‡ Department of Molecular Physiology and Biological Physics, University of Virginia, Charlottesville, Virginia; § Laboratory of Cellular and Molecular Biology, National Institutes of Health, Baltimore, Maryland; ** Department of Molecular and Cellular Biology, University of Arizona, Tucson, Arizona

**Keywords:** Edc3, P bodies, decapping, mRNA decay

## Abstract

The control of mRNA translation and degradation is mediated in part by a set of proteins that can inhibit translation and promote decapping, as well as function in the assembly of cytoplasmic mRNP granules referred to as processing bodies (P-bodies). The conserved enhancer of mRNA decapping 3 (Edc3) protein functions to promote both decapping and P-body assembly. Crystal structures of the YjeF_N domain in hEdc3 identified a putative binding site for a small molecule. Structure modeling of the human Edc3 Yjef_N along with other Yjef_N-containing proteins suggests that this molecule is related to NAD(H). We now show human Edc3 directly binds NADH. We also show that human and yeast Edc3 chemically modify NAD *in vitro*. Mutations that are predicted to disrupt the binding and/or hydrolysis of an NAD-related molecule by yeast and human Edc3 affect the control of mRNA degradation and/or P-body composition *in vivo*. This suggests that the interaction of Edc3 with an NAD-related molecule affects its function in the regulation of mRNA translation and degradation and provides a possible mechanism to couple the energetics of the cell to posttranscriptional control. Moreover, this provides a unique example of and lends strength to the postulated connection of metabolites, enzymes, and RNA.

Recent insights suggest that cytoplasmic mRNA regulation is more extensive and disease-relevant than previously appreciated (Morris *et al.* 2010). After transcription, maturation, and subsequent nuclear export, mRNAs are regulated at the level of mRNA stability, localization, and/or protein synthesis. mRNAs do not exist naturally as solitary molecules, but rather as complexes with their associated proteins, or mRNPs. It is the combination of inherent *cis*-elements of each mRNA and its associated bound protein factors that determine mRNA fate.

Translation-competent mRNAs possess the canonical 5′ ^7^mG-cap and 3′ poly(A) tail *cis*-elements and are associated with ribosomes and translation initiation factors. mRNAs entering a degradative pathway are bound by the decapping enzyme, Dcp2, its accessory proteins, and the deadenylation complex (Franks and Lykke-Andersen 2008). Concentrations of degradative machinery and its associated mRNAs are found in cytoplasmic foci termed processing bodies (P-bodies) (Franks and Lykke-Andersen 2008; Balagopal and Parker 2009).

A conserved modulator of decapping and P-body formation is the enhancer of mRNA decapping 3 (Edc3) protein. Edc3 directly binds and stimulates the decapping enzyme *in vitro* (Harigaya *et al.* 2010; Nissan *et al.* 2010; Fromm *et al.* 2011). Edc3 also affects the decay rates and/or steady-state levels of multiple mRNAs *in vivo*. For example, in yeast Edc3 affects the stability of specific mRNAs (Badis *et al.* 2004; Dong *et al.* 2007) and is required for general decapping when the decapping enzyme is partially functional (Kshirsagar and Parker 2004). In addition, microarray data from Drosophila S2 cells (Eulalio *et al.* 2007) indicate that Edc3 modulates the level and decay rates of hundreds of mRNAs. In addition to directly binding and activating Dcp2 enzymatic activity (Harigaya *et al.* 2010), Edc3 in yeast and/or humans also interacts with Dhh1/Rck, Lsm1, Scd6/Rap55, and Xrn1 (Nissan *et al.* 2010; Kshirsagar and Parker 2004; Ling *et al.* 2008; Tritschler *et al.* 2009) and is thought to play a scaffolding role in assembly of a larger decapping complex (Decker *et al.* 2007). Consistent with these results, Edc3 in yeast plays a major role in the assembly of P-bodies (Decker *et al.* 2007), although how Edc3 function is modulated remains to be determined.

An emerging theme in posttranscriptional gene regulation is a potentially widespread connection between metabolism and the regulation of mRNA function. This regulatory network has been suggested based on the observations that some metabolic enzymes have been identified as binding and being regulated by mRNAs (Castello *et al.* 2012; Hentze and Preiss 2010; Mitchell *et al.* 2013). If metabolites are involved in regulating mRNAs, then one also anticipates that some previously identified RNA-binding proteins may contain binding pockets for metabolites that would influence their function. Strikingly, the structure of the YjeF_N domain of human Edc3 (hEdc3) revealed a putative small molecule–binding pocket. Moreover, the YjeF_N domain was required for RNA-binding and dimerization of Edc3, whereas it also influenced regulation of Edc3 target mRNAs (Ling *et al.* 2008). Based on these observations, we hypothesized that Edc3 binds to a cellular metabolite in a manner that regulates its function, thereby coupling posttranscriptional regulation of gene expression to some aspect of metabolism.

The putative binding pocket of Edc3 overlapped with several YjeF_N domain–containing proteins from several species, including those with demonstrated binding to NADP and NAD (Ling *et al.* 2008). Underscoring the similarities between these binding pockets is the common presence of negatively charged invariant residues (Ling *et al.* 2008; Shumilin *et al.* 2012). YjeF_N domains are one of the most common functionally uncharacterized protein domains (Finn *et al.* 2008). They are predicted to possess conserved enzymatic activity (Anantharaman and Aravind 2004). More recently, structural analyses have revealed that YjeF_N domains frequently bind to NAD-related molecules (Shumilin *et al.* 2012), which is consistent with a report arguing that the yeast YjeF_N domain–containing protein YNL200C functions in NAD(P)(H) repair (Marbaix *et al.* 2011).

In this work, we demonstrate that the Edc3 binds NADH and mutations in the predicted binding sites affect Edc3 function. Comparative structural modeling of several Yjef_N domains suggests that the Edc3 Yjef_N domain binds a NAD(H)-related molecule. Utilizing isothermal titration calorimetry, we found that hEdc3 binds to NADH with physiological range affinity. Moreover, mutations in the putative NADH-binding pocket alter several aspects of Edc3 function in yeast and human cells, including subcellular localization, P-body formation, and the control of mRNA degradation. We also observe that purified Edc3 can modify NAD *in vitro*, indicating that Edc3 may have enzymatic activity. This identifies the interaction of Edc3 with NAD(H) as important in the regulation of mRNA translation and degradation and provides a possible mechanism to couple the energetics of the cell to posttranscriptional control. This also provides an example of a second line of evidence for the emerging coupling of metabolism to RNA control wherein RNA-binding proteins will bind and be regulated by metabolites.

## Materials and Methods

### Plasmids

Edc3-mCherry has been described (pRP1574) (Buchan *et al.* 2008). yEdc3 mCherry variants were constructed using oRP 1536 and 1537 (S351L), oRP 1538 and 1539 (K354F), oRP 1540 and 1541 (D466V), and the Quikchange II site–direction mutagenesis kit (Stratagene). His-yEdc3 (pRP 1440) has been described (Decker *et al.* 2007). oRP1708 and oRP1709 were used to clone full-length hEdc3 into pMBP-parallel1 from a cDNA clone. hEdc3-250C has been described (Ling *et al.* 2008). oRP1671 and 1672 were used to construct both hEdc3-250C-D443V (pRP 2413) and Flag-hEdc3-D443V-RFP (pRP 2414). Flag-hEdc3-RFP was a kind gift from Dr. Mark Larance (University of Dundee, Dundee, UK) (Larance *et al.* 2010). Yeast strains, plasmids, and oligos used in this study are listed in Supporting Information, Table S1.

### Antibodies

Antibodies to the following proteins were used in this study: GFP (Covance); DDX6 (Cell Signaling); PGK1 (Invitrogen); and hDcp1a and hEdc3 (a gift from Dr. Jens Lykke-Andersen) (Fenger-Gron *et al.* 2005).

### Isothermal titration calorimetry

Isothermal titration binding assays were performed at 25° using an iTC200 instrument (MicroCal). The measurements were performed in 100 mM HEPES buffer pH 7.5 with 100 mM NaCl using 50–120 μM protein solutions. The final ligand concentrations exceeded the protein concentration by 1.6- to 3-times. Data analysis was conducted with the Origin software (OriginLab).

### Cell culture

HeLa cells were maintained at 37°, 5% CO_2_, in DMEM supplemented with 10% FBS. Transfections were performed with Lipofectamine 2000 using the manufacturer’s protocol; 400 ng and 25 μg indicated Flag-RFP-hEdc3 plasmid were transfected for microscopy and immunoprecipitation experiments, respectively. Cells were harvested/analyzed ∼20 hr after transfection. IPs were performed using Flag-M2-agarose (Sigma) as described previously (Walters *et al.* 2010). HeLa cells were treated with 0.5 mM sodium arsenite for 1 hr when indicated (Kedersha *et al.* 2008).

### Microscopy

All microscopy experiments were performed using a Deltavision RT microscope system as described previously (Buchan *et al.* 2010). P-bodies were quantified in a blind manner using three independent experiments comprising at least 100 total counted granules (Buchan *et al.* 2010).

### Northern blot analysis

Northern blot analyses were performed using standard methods as previously described (Decker *et al.* 2007). A standard Student *t*-test was used to calculate P values. Probes used for individual mRNAs are listed in Table S1.

### Thin-layer chromatography

Recombinant yEdc3 and hEdc3-250C were purified as previously described (Nissan *et al.* 2010). ^32^P-radiolabeled NAD was purchased from Perkin-Elmer. NAD was converted to NADH in 0.1 M NaPO_4_, pH 7, and 10 mM EDTA using formate dehydrogenase (FDH) from *Pseudomonas* sp101. Conversion of NAD to NADH was analyzed using spectrometry at 260 and 340 nM. Reactions were performed using 5 μg recombinant protein and 0.5 μL ^32^P-NAD in a buffer of 150 mM NaCl, 50 mM Tris-HCl, pH 7.5, and 10 mM DTT for the indicated time period (1 hr if not denoted) at 30°. Reactions were then run on PEI-cellulose chromatography plates in a solvent of 0.4 M guanidine, pH 6, for one-dimensional analysis and saturated ammonium sulfate for the second dimension (Bochner and Ames 1982). ^32^P-NAD is 800 Ci/mmol, 5 mCi/mL, 6.25 μM. Radiolabeled phosphate group is 5′ alpha to the adenine.

## Results

### Comparison of the YjeF_N small molecule–binding pockets reveals common features

**Figure 1:**
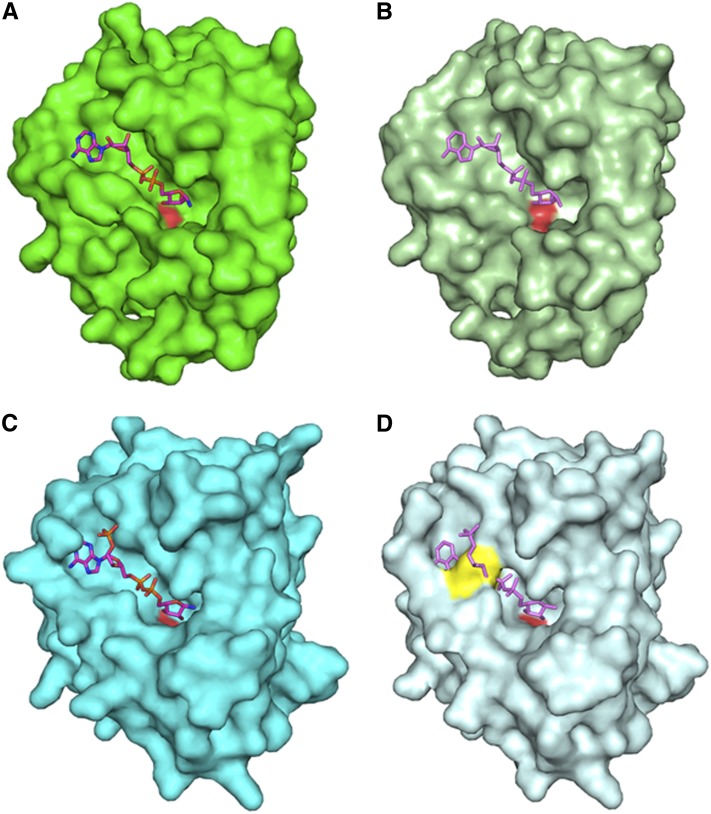
Modeling of NAD-related compounds bound to various Yjef_N domains. (A) A surface of Tm0922 subunit with bound NAD (PDB:3RSG; only Yjef_N domain is shown). (B) A surface of *apo*-Tm0922 subunit (PDB:2AX3; only Yjef_N domain is shown) with NAD superimposed from (A). (C) A surface of AI-BP subunit with bound NADP (PDB:3RNO). (D) A surface of *apo*-AI-BP subunit (PDB:2O8N) with NADP superimposed from (C). A side chain of F162 that blocks the trench is shown in yellow. The left subunit is shown as a surface with the loop β4-αD that blocks the trench compartment colored is shown in yellow. In all panels, the nicotinamide ring of bound NAD(P) disordered or hydrolyzed in the crystal structures is not shown. The carboxylate oxygen atoms of invariant aspartate (D147^Tm0922^, D188^AI-BP^) are shown in red in all surface representations.

The demonstration that YjeF_N domains often bind NAD-related compounds (Shumilin *et al.* 2012) suggested Edc3 might also bind a NAD-related molecule through its YjeF_N domain. To address this possibility, we first compared the Edc3 YjeF_N domain to other YjeF_N domains. Interestingly, in the YjeF_N domain, ligand-binding sites in AI-BP and Tm0922 consist of two compartments, a trench that runs along the protein surface and a perpendicular pocket (Figure 1) (Shumilin *et al.* 2012). In both AI-BP and Tm0922, NAD-related ligands bind in an extended conformation with their ADP moieties accommodated in the trench and nicotinamide moieties approaching an invariant aspartate residue positioned at the top of the pocket. The trench compartment is flanked by the loop β1-αA on one side and β4-αD on the other. The β4-αD loop provides the majority of polar interactions that coordinate an ADP moiety of the bound NAD(P)(H) (Shumilin *et al.* 2012). In the *apo*-structures of AI-BP and Tm0922, the width of the gap between the loops β1-αA and β4-αD is sufficient to accommodate a dinucleotide (Figure 1).

Similar to AIBP and Tm0922, the pocket compartment of a putative ligand-binding site that contains an invariant D443 is present in the *apo*-structure of the YjeF_N domain of human Edc3 (Ling *et al.* 2008). However, attempts to model NADH binding in the corresponding trench compartment of *apo*-hYjeF_N reveal an extensive steric clash between the ADP moiety of dinucleotide and the β4-αD loop that is shifted toward the β1-αA loop, closing the trench (data not shown). This clash is consistent with the possibility that Edc3 undergoes a conformational change to bind NADH.

### hEdc3 binds NADH

Recent work has demonstrated that YjeF_N domains bind compounds that contain ADP-ribose or an ADP-ribose phosphate moiety (Shumilin *et al.* 2012). Thus, we used isothermal calorimetry to characterize the interaction of hEdc3 with metabolic compounds containing these or similar fragments (ADP-ribose, NAD, NADH, NAAD, NADP, NADPH, NAADP, adenine, adenosine, AMP, ADP, ATP, and CoA).

**Figure 2:**
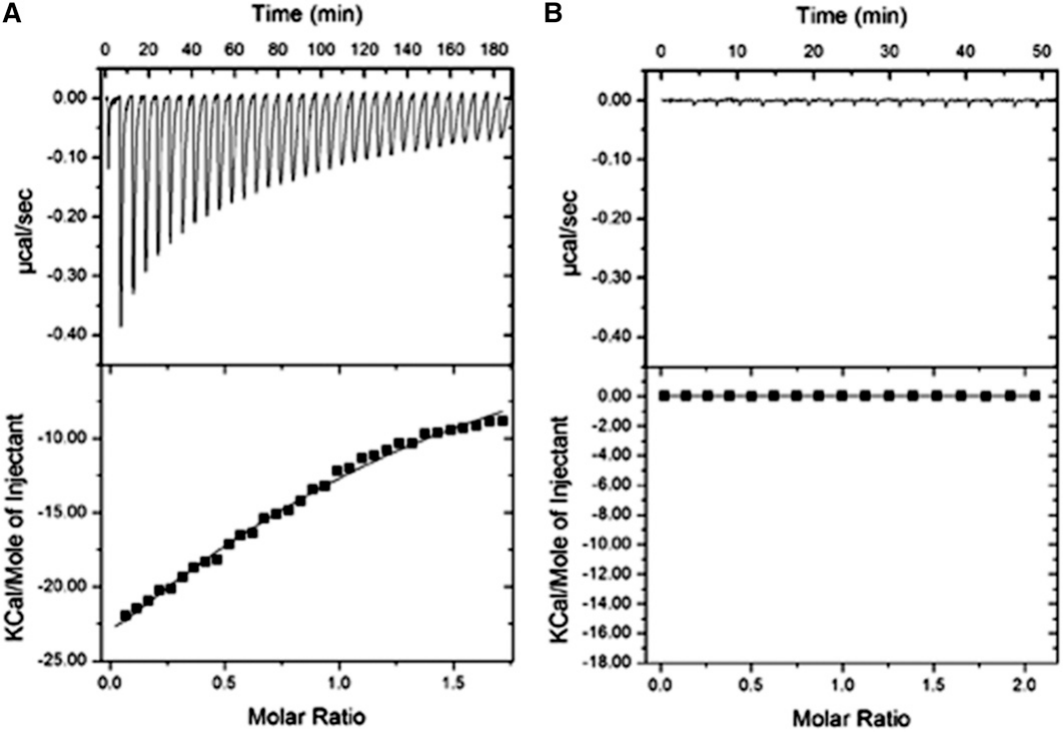
Human Edc3 binds NADH with high affinity. Isothermal titration calorimetry profiles and fitting curves for the binding of hEdc3 to NADH (A) and buffer control (B).

ITC demonstrated binding of the full-length hEdc3 to NADH but not to the other tested metabolites (Figure 2 and data not shown). The binding affinity of NADH has an equilibrium dissociation constant (K_d_) of 162.9 ± 27.4 μM, a stoichiometry (N) of 1.17 ±0.08, ΔH of −49.2 ± 6.4 kcal/mol, and ΔS of −148 cal/mol/deg. This binding affinity is well within range to bind cellular concentrations of NADH (0.2 mM) and/or NAD (1–2 mM) (Rigoulet *et al.* 2004; Belenky *et al.* 2007). There was no binding detected in the control reaction (Figure 1B). This argues that, like other YjeF_N domain proteins, Edc3 binds an NADH-related molecule, presumably through the pocket on the YjeF_N domain; however, we have been unable to stably express and purify Edc3 proteins with mutations in the predicted binding pocket at levels sufficient for ITC analyses.

### The binding pocket on the human YjeF_N domain is required for hEdc3 function

To determine the significance of the interaction between Edc3 and NAD(H), we constructed mutations in the putative binding pocket on the hEdc3 YjeF_N domain that would be predicted to alter the binding of Edc3 to NAD(H). Based on the interactions of NAD(H)-like molecules with other YjeF_N family members, we identified mutations in the human and yeast proteins (D443V and D466V, respectively) that would be expected to alter any hypothetical enzymatic activity of Edc3. Moreover, we constructed two mutations in the yeast Edc3 (S351L, K354F) that would be predicted to limit the binding of NAD(H) to Edc3.

**Figure 3:**
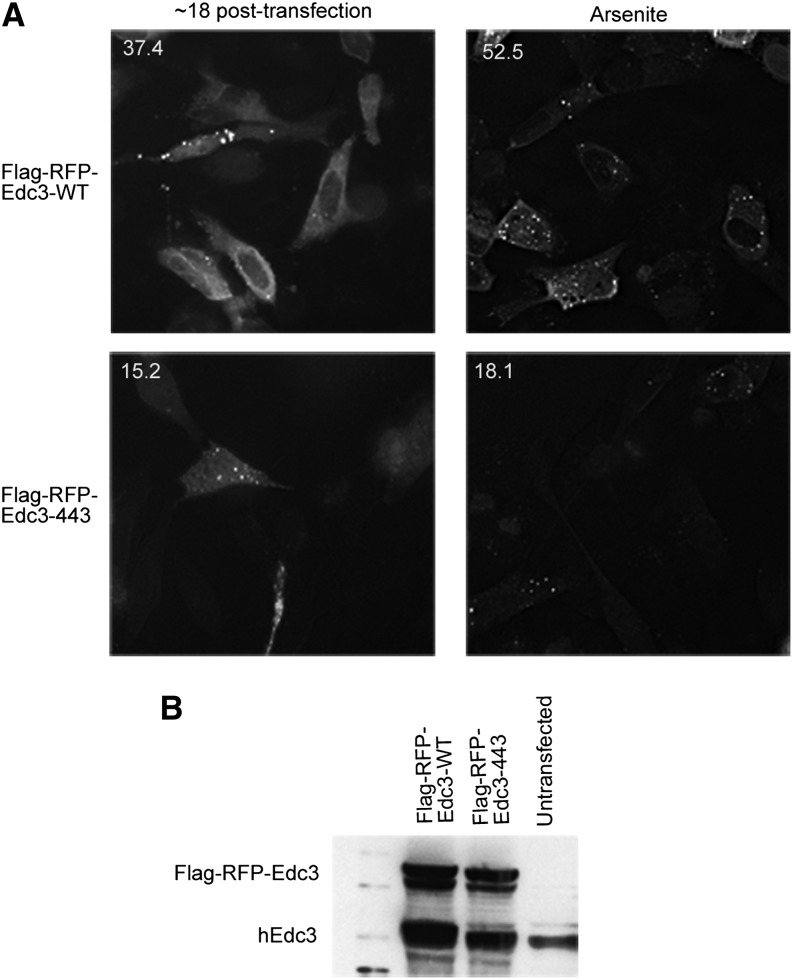
Small molecule binding alters the subcellular localization of hEdc3 variants. (A) Flag-RFP-Edc3 WT or D443V was transfected into HeLa cells and analyzed at the time indicated. In the right panels, sodium arsenite (0.5 M) was added for 1 hr. Numbers in upper left corners of each panel are the percentage of cells containing foci/total cells containing fluorescent signal and were counted over at least three independent experiments. A representative image is shown. (B) Western analysis of samples from (A) using hEdc3 antibody that recognizes both endogenous Edc3 and exogenous Flag-RFP-Edc3.

Because hEdc3 is a component of P-bodies, we first asked how mutations in the NAD(H)-binding pocket in hEdc3 affected its accumulation in P-bodies. We used Edc3 as a marker for P-bodies because of the numerous studies in yeast and mammalian cells in which this has been demonstrated (Buchan *et al.* 2008, 2010; Kedersha *et al.* 2008). For this experiment, we expressed either a WT or a D443V Flag-RFP-Edc3 fusion protein into HeLa cells and examined the subcellular location of Edc3 (Larance *et al.* 2010). As expected, we observed that the WT Edc3 accumulated in P-bodies (Figure 3A, upper left panel). However, the D443V mutant showed reduced accumulation in P-bodies (37.4% of cells with WT Edc3 foci *vs.* 15.2% for D443V) (Figure 3A, left panels), despite being expressed at similar levels to the WT protein as judged by Western analysis (Figure 3B). This effect is more pronounced when P-body formation is increased by addition of sodium arsenite (Kedersha *et al.* 2008) (52.5% for WT *vs.* 18.1% for D443V) (Figure 3A, right panels). This suggests that the binding of NAD(H) to Edc3 is required for its peak accumulation into P-bodies.

In principle, the binding of an NAD-related molecule to Edc3 could limit its accumulation in P-bodies by decreasing protein:protein interactions with other resident P-body proteins (*i.e.*, Dcp1a, Rck, itself). Alternatively, NAD^+^ binding could alter additional properties of Edc3 that decrease its ability to localize to P-bodies (*i.e.*, RNA binding). To address these possibilities, we performed coimmunoprecipitation of Flag-Edc3-RFP from sodium arsenite–treated HeLa cells and examined the ability of endogenous Edc3, Dcp1a, or DDX6 to co-purify. Interestingly, after precipitating similar amounts of each Flag-tagged construct, we observed that endogenous Edc3, Dcp1a, and DDX6 all coimmunoprecipitated with both WT and D443V Edc3 variants (Figure S1); however, a slight decrease in co-purification of endogenous Edc3 and DDX6 with D443V was detected (Figure S1). The observation that Edc3 still interacts with multiple proteins suggests it is still functional and not completely misfolded. These results suggest that NAD(H) binding is not absolutely required for individual protein–protein interactions *per se* but may primarily affect the assembly of Edc3 into P-bodies.

### Yeast Edc3 requires NAD(H) binding for proper localization

**Figure 4:**
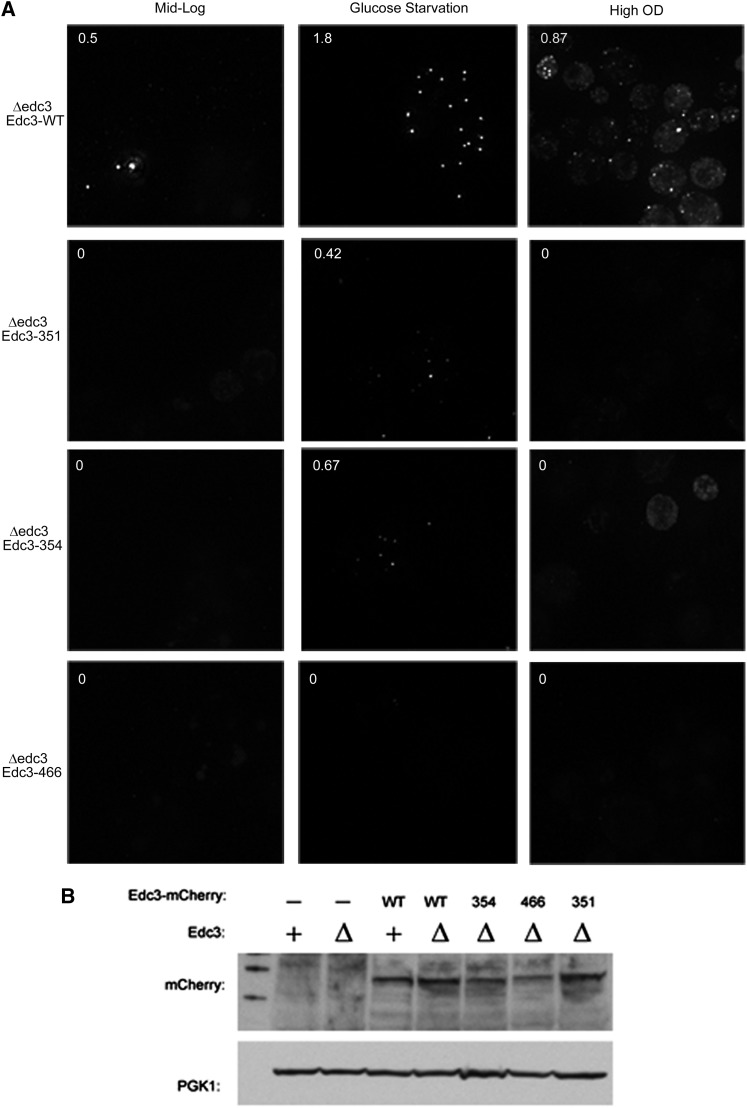
yEdc3 requires small molecule binding and/or catalysis for proper localization. Δedc3 strains were transformed with indicated yEdc3 variants and P-body formation assessed (quantified as foci/cell; upper left corner). Cells were assayed under mid-log (∼0.4 OD; left panels), glucose-starved (10 min; middle panel) or high-OD (∼2 OD; right panels) conditions. This experiment (and all microscopy experiments) was performed at least three times. (B) Western analysis of indicated yeast strains, with PGK1 serving as a loading control.

To determine if the function of the small molecule–binding pocket was conserved, we examined the effects of mutations in the yeast Edc3 protein. The aforementioned yEdc3 variants D466V, S351L, and K354F mutants were constructed in the context of an mCherry-tagged yEdc3 to allow examination of their subcellular locations. Next, we expressed each of these variants or WT Edc3 in edc3Δ strains and examined their location under mid-log, glucose starvation (10 min), or high OD (OD600 ∼2) conditions (Figure 4A). Quantification of foci-containing cells is shown in top left of each image (expressed as foci/cell).

As expected, during mid-log (nonstress) growth conditions, several WT Edc3 foci were visible (Figure 4A, top left panel). These P-bodies increased in intensity and number under both glucose starvation and high OD conditions (Figure 4A). Interestingly, the Edc3-351, 354, and 466 mutant proteins showed reduced accumulation in P-bodies constructs under mid-log or high OD conditions (Figure 4A) with a few foci that were present when cells were deprived of glucose. In these experiments, the S351L variant had the weakest phenotype (Figure 4A). One possibility for the failure of Edc3 to accumulate in P-bodies is that the Edc3 variants are simply not expressed; however, we found approximately equal expression using Western analysis (Figure 4B). From these results, we surmised that small molecule–binding alters Edc3 subcellular localization in human and yeast cells.

### yEdc3 variants reduce P-body assembly

**Figure 5:**
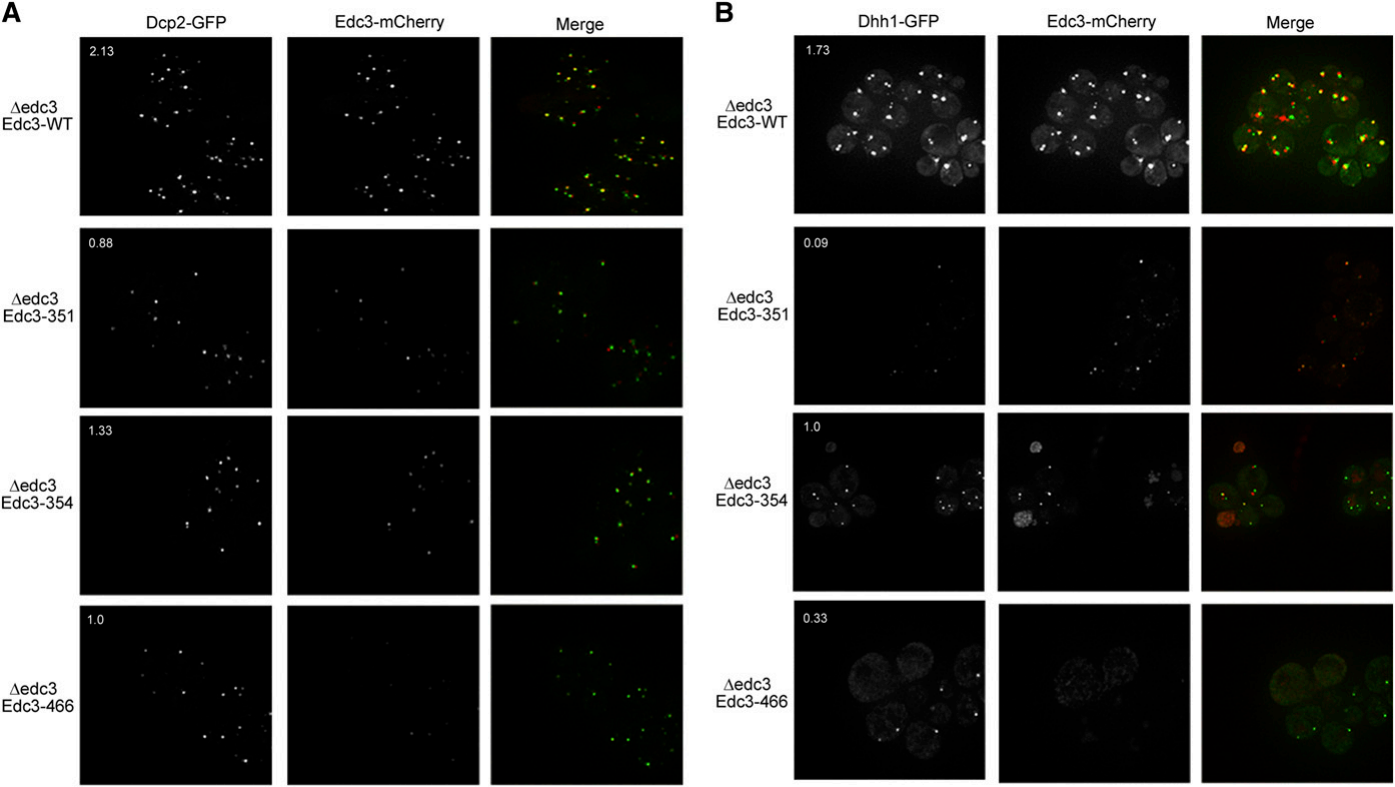
Dcp2 (A) and Dhh1 (B) accumulations in P-bodies are diminished in the presence of yEdc3 small molecule binding mutants. Experiments were performed as in Figure 3, except all cells were analyzed under glucose-starved (10 min) conditions. Quantification was performed as in Figure 3.

P-bodies contain enzymes involved in decapping and exonucleolytic decay as well as a variety of translational repressors (Balagopal and Parker 2009). In principle, the reduction in Edc3 in P-bodies attributable to mutations in the binding pocket could solely result in less Edc3 localizing to P-bodies or could also lead to reductions in other components of P-bodies. To distinguish these possibilities, we examined whether mutations in the Edc3-binding pocket also affected the accumulation of other components in P-bodies. We performed this experiment by examining Edc3 mCherry variants (or WT) in otherwise edc3Δ strains expressing Dcp2-GFP or Dhh1-GFP, two additional P-body resident proteins, under glucose deprivation (Figure 5).

As compared to WT Edc3, Dcp2-GFP and Dhh1-GFP foci were decreased in number when coexpressed with the Edc3 variants (Figure 5, A and B). The Edc3-S351L and the Edc3-D466V mutants exhibited the most pronounced decrease of Dcp2 or Dhh1 in P-bodies (Figure 5, A and B). The alternate Edc3 forms did not affect Dcp2-GFP or Dhh1-GFP expression levels (Figure S2). As previously observed, Edc3-mCherry variants did not form foci in the numbers or intensity as did those of WT (Figure 5, A and B). We concluded from this set of experiments that lack of small ligand binding or catalysis disrupts not only Edc3 localization to P-bodies but also the accumulation of other P-body components, which is consistent with previous work showing that Edc3 plays an important assembly role for yeast P-bodies (Decker *et al.* 2007).

### mRNA regulation by Edc3 is affected by alterations in the small molecule binding site

In yeast cells, two mRNAs, Rps28B (a ribosomal protein mRNA) and YRA-1 (a nuclear export factor), have been described to date as being dependent on Edc3 for their mRNA decapping events (Badis *et al.* 2004; Dong *et al.* 2007). We reasoned that small molecule binding may alter Edc3-mediated regulation of these mRNAs because small molecule binding altered the subcellular localization of both Edc3 and other P-body resident proteins (Figure 4 and Figure 5), and that the YjeF_N domain is required for efficient mRNA binding (Ling *et al.* 2008). Accordingly, we assessed how the mutations in the Edc3-binding pocket affected Rps28B and pre-YRA1 mRNA degradation.

**Figure 6:**
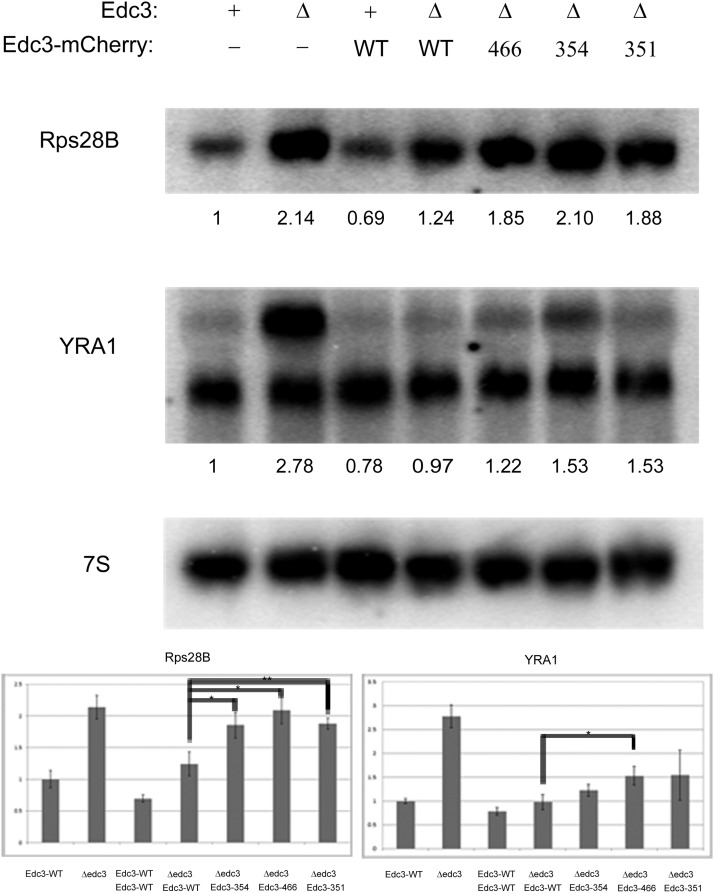
yEdc3 mRNA regulation is lost concurrent with small molecule binding and/or catalysis. Northern blot analysis was performed on indicated yeast strains during mid-log growth conditions. Blots were probed for Rps28B or YRA1, with 7S used for loading. Quantification (after 7S normalization) is shown below respective blots. Northern blot analyses were performed in quadruplicate and error bars represent 1 SD. A representative blot is depicted. *P value < 0.005; **P value < 0.0001.

We analyzed steady-state mRNA levels of Rps28B and YRA-1 under mid-log growth conditions, again complementing edc3Δ strains with WT or aforementioned variants (Figure 6). As previously shown (Badis *et al.* 2004; Dong *et al.* 2007), the levels of Rps28B and YRA-1 pre-mRNA (top band) increased in edc3Δ strains (Figure 6). Once this strain was complemented with WT Edc3, the amount of these mRNAs returned to approximate parental strain levels (Figure 6). The D466V variant failed to modulate Rps28B and YRA-1, whereas the K354F and S351L variants solely impacted Rps28B regulation (Figure 6). This divergence among variants is consistent with our microscopy data in which the D466V mutation has the most pronounced effect (Figure 4 and Figure 5), and it also suggests that the specific mechanism by which Edc3 accelerates the decay of these two mRNAs may be slightly different. Nevertheless, the critical observation is that mutations in the NAD(H)-binding pocket affect the ability of Edc3 to promote mRNA degradation of the Rps28B and pre-YRA1 mRNAs.

### Yeast and hEdc3 modify NAD *in vitro*

In principle, Edc3 could simply bind NAD-related molecules and could be influenced by that binding in an allosteric manner. Alternatively, as suggested by the putative catalytic residues in the potential YjeF_N domain-binding pocket, Edc3 might act as an enzyme to chemically modify NAD(H) and/or use NAD(H) to modify other factors. A precedent for this type of function comes from the study of sirtuins, which utilize NAD to act as protein deacetylases (Haigis and Sinclair 2010). To determine if Edc3 has any enzymatic activity on NAD, we incubated recombinant Edc3 with radiolabeled NAD or a mixture of NAD and NADH (NAD(H)). These reactions were then analyzed using thin-layer chromatography (Bochner and Ames 1982). For this, yeast and human putative catalytic mutants were cloned and purified along with full-length yEdc3 (Nissan *et al.* 2010) and the hEdc3 YjeF_N domain (250C) (Ling *et al.* 2008), respectively. The construct containing solely the Yjef_N domain of hEdc3 was chosen for these experiments because the D443V could be purified in sufficient quantities in this form for these particular experiments.

**Figure 7:**
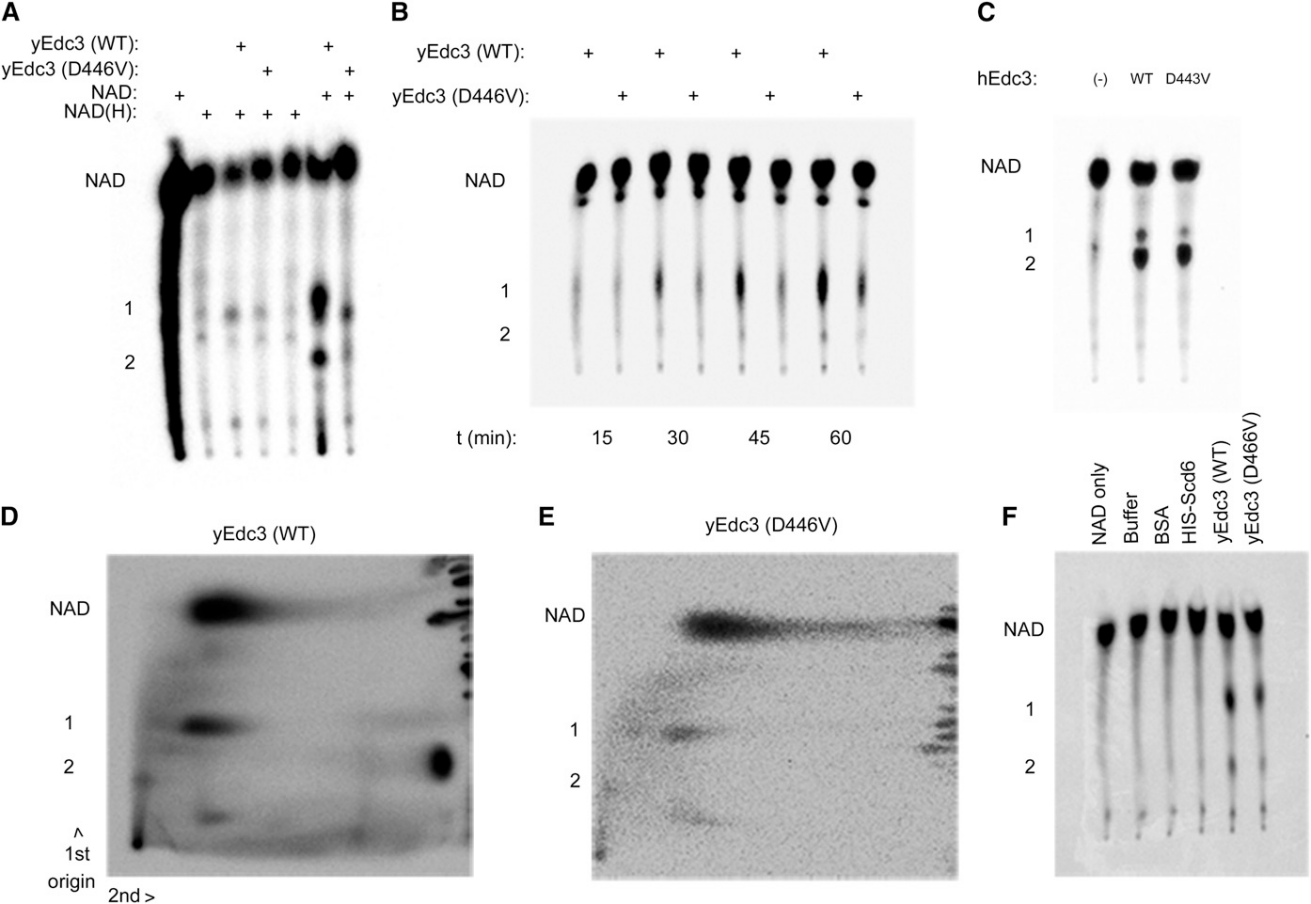
Yeast and human Edc3 modify NAD *in vitro*. (A) ^32^P-NAD or a mixture of ^32^P-NAD and NADH was incubated with full-length recombinant yeast Edc3 and assayed using TLC after 2 hr. All panels are labeled similarly as follows: products 1 and 2 and full-length NAD. (B) Time course of ^32^P-NAD and recombinant yeast Edc3 or D446V. (C) Reactions performed with ^32^P-NAD and hEdc3 Yjef_N WT or D443V. (D) 2D TLC analysis (after 2-hr incubation) of ^32^P-NAD incubated with WT yeast Edc3. (E) As in (D) but with yEdc3 D466V. (F) As in (A) but with additional controls.

Incubation of yeast WT Edc3 with radiolabeled NAD gave rise to two slower migrating species that were not found in reactions containing only NAD (Figure 7A, second lane from right, labeled 1 and 2). Moreover, these products were not observed when radiolabeled NAD was incubated with BSA or other similarly purified recombinant proteins (Figure 7F). The D466V variant gave rise to similar species, but with far less intensity (Figure 7A, compare two rightmost lanes). A time course analysis revealed that accumulation of these products begins after 30 min of incubation and was slower in the mutant than in the WT Edc3 (Figure 7B). This suggests that Edc3 can modify NAD and that it does so in a manner that requires the binding pocket in the YjeF_N domain. We next examined the migration pattern of these products using 2D TLC using WT yEdc3 and NAD (Bochner and Ames 1982). Product 1 migrated similarly to NAD in the second dimension, whereas product 2 migrated over a much greater distance, suggesting a difference in base (Figure 7D) (Bochner and Ames 1982). We obtained similar results using the hEdc3 YjeF_N domain (250C) (Ling *et al.* 2008) (Figure 7C), although the differences between WT and D443V are not as apparent as in the yeast constructs. Thus, we concluded that human and yeast Edc3 can modify NAD, although whether Edc3 directly functions as an enzyme *in vivo* remains to be demonstrated.

## Discussion

Several lines of evidence now indicate that Edc3 interacts with an NAD-related ligand. This possibility was first suggested by the presence of a putative ligand-binding pocket in the YjeF_N domain of Edc3 (Ling *et al.* 2008). Two other members of the YjeF_N protein family were shown to interact with ligands structurally similar to NAD (Shumilin *et al.* 2012; Marbaix *et al.* 2011). These proteins, mouse apolipoprotein A-I–binding protein (AI-BP) and Tm0922 from *Thermotoga maritime*, share a number of common features with the YjeF_N domain of Edc3, including a similar fold and location of an invariant aspartate residue (D443^hEdc3^/D466^yEdc3^). This residue has been suggested to play a possible role in catalysis (Shumilin *et al.* 2012). Structural modeling of several Yjef_N domains is consistent with the Edc3 Yjef_N domain binding a NAD(H)-related compound (Figure 1). Direct evidence for Edc3-binding NAD-related molecules is that full-length hEdc3 protein binds to NADH *in vitro* (Figure 2).

Our data suggest that Edc3 can chemically alter NAD because its incubation with either human or yeast Edc3 leads to chemical alteration of NAD in a manner dependent on the ligand-binding pocket (Figure 7). This is consistent with an observation that in every determined complex structure of related proteins, the nicotinamide ring of the NAD-related ligand is not visible in the electron density because of hydrolysis or disorder (Shumilin *et al.* 2012). Thus, although we cannot distinguish precisely what chemical form of NAD binds to Edc3 *in vivo*, we conclude that Edc3 binds to NAD or NAD-related molecules *in vivo* and can chemically modify that compound, although whether any enzymatic activity of Edc3 is required for its function *in vivo* remains to be determined.

Our evidence also indicates that the interaction of Edc3 with an NAD-related ligand is required for optimal Edc3 function *in vivo* in yeast and human cells. First, mutations in the predicted ligand-binding pocket in human Edc3 reduce the accumulation of Edc3 in P-bodies in HeLa cells (Figure 3). Similarly, mutations in the yeast Edc3 ligand pocket reduce Edc3 accumulation in P-bodies (Figure 4) and the accumulation of Dcp2 and Dhh1 in yeast P-bodies (Figure 5). Finally, mutations in the yeast Edc3 pocket affect the turnover of the Rps28B and pre-YRA1 mRNAs (Figure 6), which are known targets of Edc3-mediated decapping (Badis *et al.* 2004; Dong *et al.* 2007).

An unresolved issue is the function of the proposed NAD(H)–Edc3 interaction. One possibility is that NAD acts as an allosteric regulator of Edc3 protein–protein or protein–RNA interactions. However, we consider this possibility unlikely because Edc3 can modify NAD *in vitro* in a manner dependent on mutations that affect function *in vivo*, and we have been unable to document any effect of NAD on Edc3 dimerization or Edc3-RNA interactions with purified proteins (data not shown). An alternative, and potentially overlapping, possibility is that Edc3 chemically converts NAD and, in that process, either creates a metabolite that affects mRNA control or, analogous to sirtuins, modifies a protein to influence its activity.

In the broadest sense, in binding NAD(H) there exist a few possibilities for Edc3: NAD(H) processing enzyme; sirtuin; or ADP-ribosyltransferase. Intriguingly, it was recently shown that YjeF_N proteins can be involved in the NADH salvage pathway via interconversion of NADHX (a hydrated form of NADH) epimers [*i.e.*, (S) to (R) form and vice versa] (Marbaix *et al.* 2011). Accordingly, it is possible that Edc3 also participates in NAD(H) salvage pathways and this activity is intimately linked to its role in mRNA decay. However, *in vitro* assays using purified hEdc3 and NADH clearly demonstrate that Edc3 does not convert NADH to NADHX (unpublished results). Future work addressing the specific biochemistry of Edc3 on NAD(H) and how this is used to coordinate mRNA regulation and cellular metabolism will be of high interest.

An important broader contribution of this study is to provide a second type of evidence that metabolism and mRNA regulation are intimately coupled. The coupling of metabolism and RNA regulation has already been suggested by observations that numerous metabolic enzymes bind and regulate mRNAs (Hentze and Preiss 2010). We now demonstrate that a known mRNA-binding protein that controls decapping is functionally modulated by the common metabolite NAD(H). We predict that additional mRNA-binding proteins will also bind and be regulated by metabolites, increasing the breadth of the mRNA–metabolite regulatory network. Consistent with this possibility, recent work has shown that the Nro1 protein or Ett1 protein, both of which regulate translation termination in *S. pombe* and *S. cerevisiae*, respectively, has a binding site for an unknown ligand and that ligand-binding site is required for Ett1 function in translation termination (Rispal *et al.* 2011). An important goal of future work will be to determine the breadth of this regulatory network and its impact on posttranscriptional gene regulation.

## Supplementary Material

Supporting Information
