# Supplementary material for: Edc3 Function in Yeast and Mammals Is Modulated by Interaction with NAD-Related Compounds
Source: G3 (Bethesda). 2014 Feb 5;4(4):613–22. doi: 10.1534/g3.114.010470 (PMC4059234; doi:10.1534/g3.114.010470)
Supplement: Supporting Information [file supp_4_4_613__index.html]

Edc3 Function in Yeast and Mammals Is Modulated by Interaction with NAD-Related Compounds — Supporting Information 

# Edc3 Function in Yeast and Mammals Is Modulated by Interaction with NAD-Related Compounds

## Supporting Information for Walters *et al.*, 2014

**Files in this Data Supplement:**

- Supporting Information - Figures S1-S2 and Table S1 (PDF, 494 KB)
- Figure S1 - Co-immunoprecipitation of hEdc3 binding proteins. (PDF, 350 KB)
- Figure S2 - Immunoblot for Dcp2-GFP and Dhh1-GFP using an antibody to GFP. (PDF, 407 KB)
- Table S1 - Yeast strains, plasmids, and oligos used in this study (.xlsx, 12 KB)
