# Supplementary material for: Edc3 Function in Yeast and Mammals Is Modulated by Interaction with NAD-Related Compounds
Source: G3 (Bethesda). 2014 Feb 5;4(4):613–22. doi: 10.1534/g3.114.010470 (PMC4059234; doi:10.1534/g3.114.010470)
Supplement: Supporting Information [file supp_g3.114.010470_010470SI.pdf]

G3: Genes | Genomes | Genetics

Edc3 function in yeast and mammals is modulated by interaction with NAD-related compounds

Robert W. Walters[*,†], Igor A. Shumilin[‡], Je-Hyun Yoon[§], Wladek Minor[‡], and Roy Parker,[*,¶]
Department Chemistry and Biochemistry[*], Howard Hughes Medical Institute , University of Colorado, Boulder, Colorado; Department of Molecular Physiology and Biological Physics, University of Virginia, Charlottesville, Virginia[‡]; Laboratory of Cellular and Molecular Biology, National Institutes of Health, Baltimore, Maryland[§]; Department of Molecular and Cellular Biology, University of Arizona, Tucson, AZ[†]

[¶]To whom correspondence should be addressed,
Roy Parker
University of Colorado
3415 Colorado Ave., JSCBB B414
Boulder, CO 80303-0596
Phone: 303 735 7780
Fax: 303 492 8425
Roy.Parker@colorado.edu

DOI: 10.1534/g3.114.010470

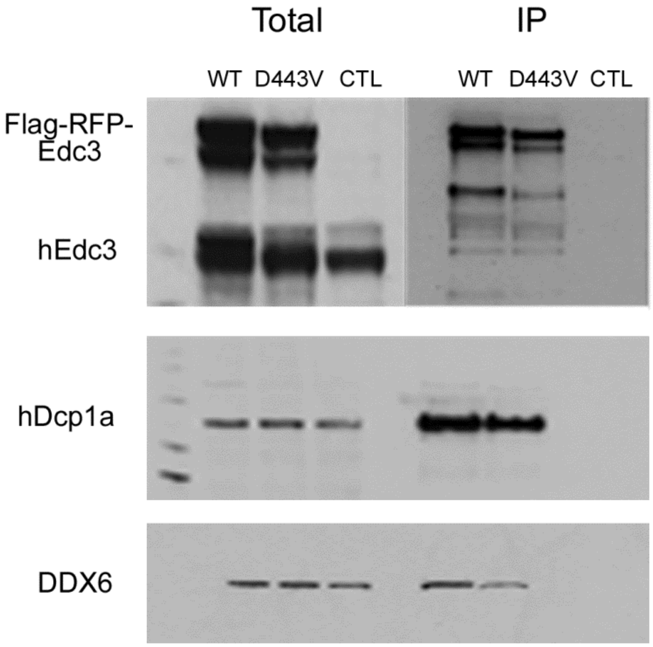

Figure S1 Co-immunoprecipitation of hEdc3 binding proteins. Flag-RFP-Edc3 WT or D443V was transfected into Hela cells and treated with sodium arsenite as described in Figure 2. Cell lysates were immunoprecipitated and probed with indicated antibodies.

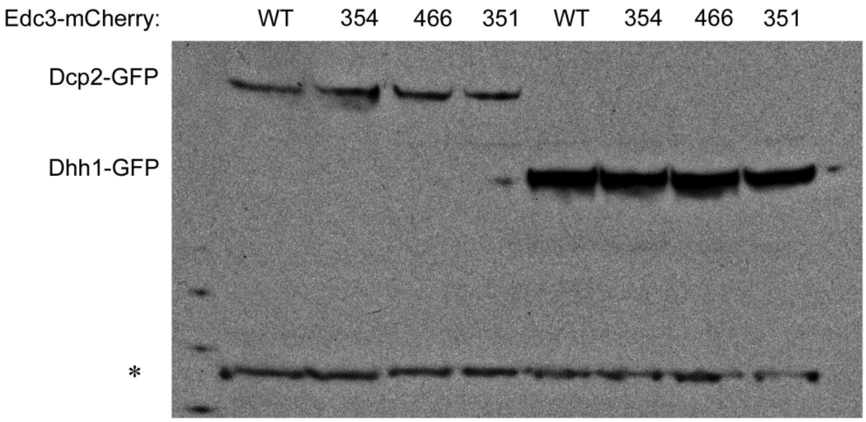

Figure S2 Immunoblot for Dcp2-GFP and Dhh1-GFP using an antibody to GFP. A non-specific cross-reactive lower band (asterisk) was used as a loading control.

Table S1 Yeast strains, plasmids, and oligos used in this study

Available for download as an Excel file at http://www.g3journal.org/lookup/suppl/doi:10.1534/g3.114.010470/-/DC1
