# Supplementary material for: Edc3 Function in Yeast and Mammals Is Modulated by Interaction with NAD-Related Compounds
Source: G3 (Bethesda). 2014 Feb 5;4(4):613–22. doi: 10.1534/g3.114.010470 (PMC4059234; doi:10.1534/g3.114.010470)
Supplement: Supporting Information [file supp_g3.114.010470_FigureS1.pdf]

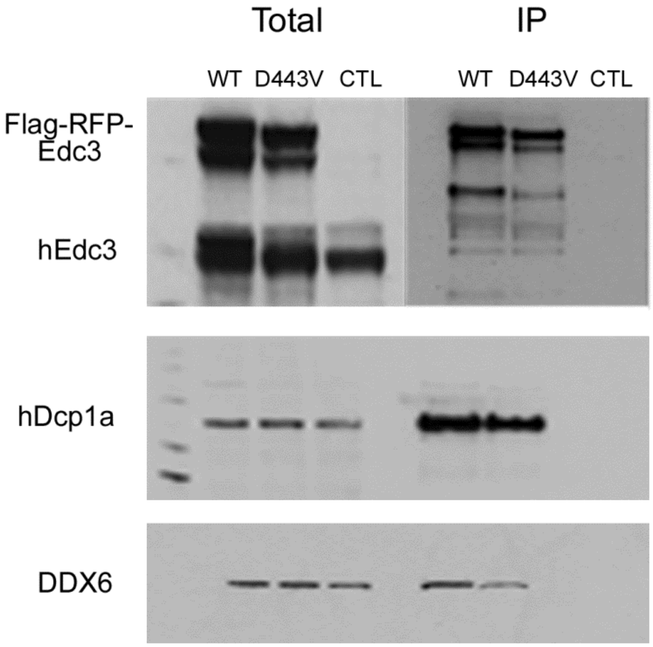

Figure S1 Co-immunoprecipitation of hEdc3 binding proteins. Flag-RFP-Edc3 WT or D443V was transfected into Hela cells and treated with sodium arsenite as described in Figure 2. Cell lysates were immunoprecipitated and probed with indicated antibodies.
