# Supplementary material for: Edc3 Function in Yeast and Mammals Is Modulated by Interaction with NAD-Related Compounds
Source: G3 (Bethesda). 2014 Feb 5;4(4):613–22. doi: 10.1534/g3.114.010470 (PMC4059234; doi:10.1534/g3.114.010470)
Supplement: Supporting Information [file supp_g3.114.010470_FigureS2.pdf]

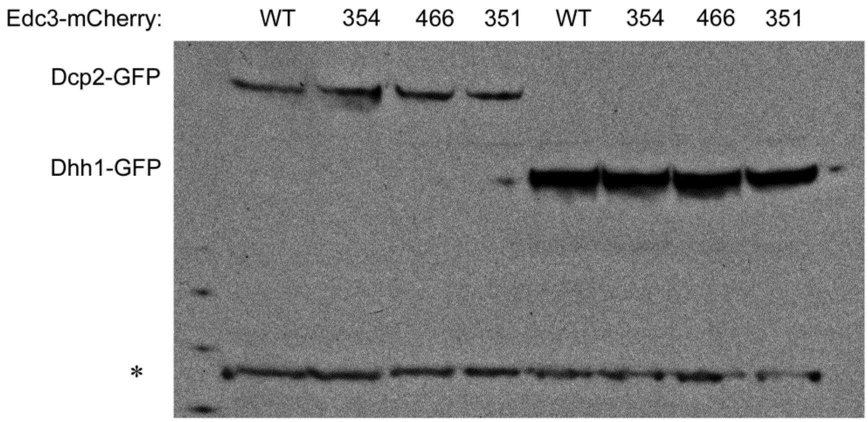

Figure S2 Immunoblot for Dcp2-GFP and Dhh1-GFP using an antibody to GFP. A non-specific cross-reactive lower band (asterisk) was used as a loading control.
